# Supplementary material for: Identification of an allosteric binding site on the human glycine transporter, GlyT2, for bioactive lipid analgesics
Source: eLife. 2019 Oct 17;8:e47150. doi: 10.7554/eLife.47150 (PMC6797481; doi:10.7554/eLife.47150)
Supplement: Supplementary file 1. [file elife-47150-supp1.docx]

**Supplementary File 1. IC_50_ values and % max. inhibition for mutant glycine transporters**

| **Mutation** | **Compound** | **IC_50_ (nM)** | **% Max. inhibition** |
| --- | --- | --- | --- |
| **GlyT2 mutants** | | | |
| **I545L** | oleoyl l-Valine | >10 µM**^a^** (4) | 22.5 ± 8.0**^b^** (p=0.0001) |
|  | oleoyl l-Aspartate | >10 µM**^a^** (3) | 4.9 ± 6.4 (p=0.0001) |
|  | oleoyl l-Tryptophan | >10 µM**^a^** (3) | 20.2 ± 1.7 (p=0.0001) |
|  | oleoyl l-Lysine | 443 (270 – 804) (7) | 80.7 ± 4.8 (p=0.8224) |
| **Y550L** | N-oleoyl Glycine | 829 (65.0 – 4170) (4) | 34.8 ± 9.1 (p=0.0011) |
|  | oleoyl L-Carnitine | >3 µM**^a^** (3) | 17.3 ± 1.4 (p=0.0001) |
|  | oleoyl l-Valine | 2930 (1680 – 5130) (4) | 57.0 ± 5.1 (p=0.0028) |
|  | oleoyl l-Leucine | 437 (214 – 8920) (3) | 41.1 ± 6.2* (p=0.0115) |
|  | oleoyl l-Serine | >3 µM**^a^** (3) | 11.2 ± 1.6 (p=0.0001) |
|  | oleoyl l-Aspartate | >3 µM**^a^** (3) | 7.0 ± 0.8**^b^** (p=0.0001) |
|  | oleoyl l-Tryptophan | >3 µM**^a^** (4) | 16.5 ± 1.7 (p=0.0001) |
|  | oleoyl l-Arginine | >3 µM**^a^** (4) | 9.3 ± 1.4 (p=0.0001) |
|  | oleoyl l-Lysine | 210 (78.6 – 559) (4) | 48.2 ± 4.1 (p=0.0001) |
|  | oleoyl d-Lysine | >3 µM**^a^** (3) | 38.4 ± 10.0 (p=0.0010) |
| **P561S** | N-oleoyl Glycine | 564 (245 – 1300) (4) | 62.1 ± 4.8 (p=0.9242) |
|  | oleoyl L-Carnitine | >3 µM**^a^**  (3) | 31.6 ± 5.2 (p=0.0001) |
|  | oleoyl l-Valine | 753 (455 – 1250) (4) | 94.5 ± 4.7 (p=0.9462) |
|  | oleoyl l-Serine | 580 (276 – 1220) (3) | 80.3 ± 7.3 (p=0.7241) |
|  | oleoyl l-Aspartate | 2540 (1490 – 4350) (3) | 58.2 ± 4.7 (p=0.0954) |
|  | oleoyl l-Tryptophan | 318 (153 – 663) (4) | 61.4 ± 4.2 (p=0.0001) |
|  | oleoyl l-Lysine | 69.2 (31.8 – 151) (5) | 71.5 ± 4.8 (p=0.0758) |
|  | oleoyl d-Lysine | 558 (247 – 1260) (3) | 76.7 ± 8.1 (p=0.4299) |
| **W563L** | N-oleoyl Glycine | 755 (317 – 1800) (3) | 46.6 ± 4.0* (p=0.0449) |
|  | oleoyl L-Carnitine | >3 µM**^a^**  (3) | 34.7 ± 3.2 (p=0.0001) |
|  | oleoyl l-Valine | 7510 (1090 – 52000) (3) | 56.3 ± 9.1**^b^** (p=0.5737) |
|  | oleoyl l-Serine | 661 (221 – 1980) (3) | 62.3 ± 5.3 (p=0.3662) |
|  | oleoyl l-Aspartate | 1160 (477 – 2800) (5) | 74.9 ± 7.5 (p=0.9965) |
|  | oleoyl l-Tryptophan | >3 µM**^a^**  (4) | 42.9 ± 2.6 (p=0.0001) |
|  | oleoyl l-Lysine | 175 (110 – 277) (4) | 73.5 ± 3.3 (p=0.1479) |
|  | oleoyl d-Lysine | >3 µM**^a^**  (3) | 42.1 ± 7.7 (p=0.0017) |
| **L569F** | N-arachidonyl Glycine | 3590 (430 – 30200) (4) | 63.7 ± 19.0 (p=0.9990) |
|  | N-oleoyl Glycine | >10 µM**^a^**  (3) | 32.5 ± 3.6 (p=0.0005) |
|  | oleoyl L-Carnitine | 1200 (267 – 5360) (3) | 45.5 ± 7.0**^b^** (p=0.0038) |
|  | oleoyl l-Lysine | 301 (157 – 577) (3) | 76.8 ± 5.8 (p=0.4504) |
| **R439L** | oleoyl l-Tryptophan | 109 (53.7 – 221) (5) | 75.3 ± 4.2 (p=0.1040) |
| **V432A** | N-arachidonyl Glycine | >10 µM**^a^**  (5) | 4.2 ± 3.2 (p=0.0018) |
|  | N-oleoyl Glycine | >10 µM**^a^**  (4) | 1.9 ± 3.2**^b^** (p=0.0001) |
|  | oleoyl L-Carnitine | >3 µM**^a^**  (4) | 6.0 ± 3.2 (p=0.0001) |
|  | oleoyl l-Lysine | 68.4 (50.8 – 92.1) (3) | 71.9 ± 1.9 (p=0.0001) |
|  | C16 ω3 Glycine | >10 µM**^a^**  (5) | 1.0 ± 3.0 (p=0.0193) |
|  | C16 ω7 Glycine | >10 µM**^a^**  (3) | 44.2 ± 6.3**^b^** (p=0.0009) |
| **F428A** | N-arachidonyl Glycine | 14400 (3540 – 58600) (4) | 64.7 ± 6.6**^b^** (p=0.9998) |
|  | N-oleoyl Glycine | >10 µM**^a^**  (4) | 25.4 ± 4.9 (p=0.0001) |
|  | oleoyl L-Carnitine | >3 µM**^a^**  (4) | 22.7 ± 3.0**^b^** (p=0.0001) |
|  | oleoyl l-Lysine | >3 µM**^a^**  (3) | 39.2 ± 2.3**^b^** (p=0.0001) |
|  | C18 ω8 Glycine | >10 µM**^a^***  (4) | 13.2 ± 2.7 (p=0.0001) |
|  | C18 ω10 Glycine | 776 (83.8 – 7180) (3) | 25.3 ± 5.3 (p=0.0092) |
|  | C16 ω7 Glycine | 5760 (2220 – 1490) (3) | 56.5 ± 7.8 (p=0.0034) |
|  | C16 ω11 Glycine | 4810 (2620 – 8830) (3) | 103 ± 7.7 (p=0.6075) |
|  | C14 ω5 Glycine | >30 µM**^a^**  (3) | 33.4 ± 3.0**^b^** (p=0.0007) |
| **GlyT1 mutant** | | | |
| **L425I** | N-arachidonyl Glycine | >10 µM**^a^** (3) | 31.6 ± 6.9 (p=0.0361) |
|  | N-oleoyl Glycine | >10 µM**^a^** (3) | 28.5 ± 5.3**^b^** (p=0.0564) |
|  | oleoyl L-Carnitine | 195 (92.4 – 411) (3) | 74.6 ± 5.3 (p=0.0024) |
|  | oleoyl l-Lysine | 236 (140 – 398) (3) | 95.6 ± 5.0 (p=0.0018) |
|  | oleoyl l-Tryptophan | >3 µM**^a^** (3) | 35.7 ± 13.2 (p=0.2787) |

Compounds were tested for inhibition of glycine transport on *Xenopus laevis* oocytes expressing mutant GlyT1 and GlyT2 transporters. n values are in brackets, with measurements taken from at least 2 batches of oocytes. Data presented are mean and 95% confidence intervals or mean ± SEM.

**^a^**Where significant inhibition was not reached, IC_50_ are presented as greater than the maximum concentration of each compound applied.

**^b^**Where inhibition was insignificant and could not be accurately calculated by curve fitting values given are the % inhibition at the highest concentration of inhibitor used.

p values are represented from one way ANOVA tests or two-tailed t-tests as described in Methods.
