# Supplementary material for: Identification of an allosteric binding site on the human glycine transporter, GlyT2, for bioactive lipid analgesics
Source: eLife. 2019 Oct 17;8:e47150. doi: 10.7554/eLife.47150 (PMC6797481; doi:10.7554/eLife.47150)
Supplement: Supplementary file 2. [file elife-47150-supp2.docx]

| **Supplementary File 2. Summary of acyl amino acids, their activity on WT transporters, and their effects in MD simulations and electrophysiological recordings of mutant transporters.** | | | | | |
| --- | --- | --- | --- | --- | --- |
| **Compound** | **Structure** | **GlyT2**  **IC_50_ (nM)** | **GlyT1**  **IC_50_ (µM)** | **GlyT2**  **% max. inhibition** | **Proposed interactions with GlyT2** |
| oleoyl l-Lysine |  | 25.5 (17.2 – 37.7) (4) | >3 | 86.8 ± 2.4 | **Residues identified by MD:** *Tail:* Val214 (TM1), Pro429 (TM5), Val432 (TM5), Ile524 (TM7), Ile545 (EL4), Phe567 (TM8) *Backbone:* Val523 (TM5), Met527 (TM7), Arg531 (EL4), Tyr550 (EL4) *Sidechain:* Trp563 (TM8)  **Mutations affecting inhibition in vitro:** *Reduces sensitivity:* F428A (TM5), V432A (TM5), Y550L (EL4) *Introduces sensitivity:* L425I (GlyT1) (EL4) *Unaffected:* I545L (EL4), W563L (TM8) |
| oleoyl l-Tryptophan |  | 54.6 (31.4 – 95.8) (4) | >10 | 86.2 ± 3.8 | **Residues identified by MD:** *Tail:* Val214 (TM1), Leu436 (TM5), Met527 (TM7), Phe567 (TM8), Ile545 (EL4) *Backbone:* Phe526 (TM7), Arg531 (EL4), Tyr550 (EL4) *Sidechain:* Arg439 (TM5), Thr555 (EL4), Trp563 (TM8)  **Mutations affecting inhibition in vitro:** *Reduces sensitivity:* I545L (EL4), Y550L (EL4), P561S (TM8) W563L (TM8) *Unaffected:* R439L (TM5), L425I (GlyT1) (EL4) |
| oleoyl l-Serine |  | 496 (308 – 799) (3) | >3 | 73.1 ± 5.1 | **Residues identified by MD:** *Tail:* Leu436 (TM5), Leu433 (TM5), Tyr550 (EL4), Trp563 (TM8), Phe567 (TM8),  *Backbone:* Arg439 (TM5), Phe526 (TM7), Tyr550 (EL4) *Sidechain:* Leu557 (EL4), Pro558 (EL4)  **Mutations affecting inhibition in vitro:** *Reduces sensitivity:* Y550L (EL4) *Unaffected:* P561S (TM8), W563L (TM8) |
| oleoyl l-Leucine |  | 143 (70.7 – 287) (4) | >10 | 72.2 ± 3.8 | **Residues identified by MD:** *Tail:* Trp215 (TM1), Leu433 (TM5), Leu437 (TM5), Ile545 (EL4), Met570 (TM8) *Backbone:* Tyr550 (EL4), Leu557 (EL4) *Sidechain:* Val523 (TM7)  **Mutations affecting inhibition in vitro:** *Reduces sensitivity:* Y550L (EL4) |
| oleoyl l-Valine |  | 1170 (643 – 2120) (4) | >30 | 90.9 ± 5.9 | **Mutations affecting inhibition in vitro:** *Reduces sensitivity:* I545L (EL4) Y550L (EL4) *Unaffected:* P561S (TM8), W563L (TM8) |
| oleoyl l-Carnitine |  | 340 (210 – 530) (3) | >10 | 71.5 ± 5.7 | **Mutations affecting inhibition in vitro:** *Reduces sensitivity:* F428A (TM5), V432A (TM5), I545L (EL4), Y550L (EL4), P561S (TM8), W563L (TM8), L569F (TM8)  *Introduces sensitivity:* L425I (GlyT1) (EL4) |
| oleoyl d-Lysine |  | 48.3 (37.2 – 62.7)  (3) | >3 | 91.0 ± 1.9 | **Mutations affecting inhibition in vitro:** *Reduces sensitivity:* Y550L (EL4), W563L (TM8) *Unaffected:* P561S (TM8) |
| oleoyl l-Arginine |  | 47.9 (27.2 – 84.2) (3) | >3 | 87.2 ± 3.7 | **Mutations affecting inhibition in vitro:** *Reduces sensitivity:* Y550L (EL4) |
| oleoyl l-Aspartate |  | 865 (468 – 1600) (4) | >10 | 76.7 ± 5.3 | **Mutations affecting inhibition in vitro:** *Reduces sensitivity:* I545L (EL4) Y550L (EL4) *Unaffected:* P561S (TM8), W563L (TM8) |
| N-arachidonyl Glycine |  | 9100 (4500 – 18000) (4) | >10 | 79.6 ± 2.1 | **Mutations affecting inhibition in vitro:** *Reduces sensitivity:* V432A (TM5), I545L (EL4),  *Unaffected:* F428A (TM5), L569F (TM8) |
| N-oleoyl Glycine |  | 500 (230 – 1100) (4) | >10 | 66.8 ± 2.8 | **Mutations affecting inhibition in vitro:** *Reduces sensitivity:* F428A (TM5), V432A (TM5), I545L (EL4), Y550L (EL4), W563L (TM8), L569F (TM8) *Unaffected:* L425I (GlyT1) (EL4), P561S (TM8) |
| C18 ω8 Glycine |  | 320 (180 – 570) (3) | >10 | 61.3 ± 1.7 | **Mutations affecting inhibition in vitro:** *Reduces sensitivity:* F428A (TM5) |
| C18 ω10 Glycine |  | 340 (140 – 840) (3) | >10 | 52.4 ± 2.2 | **Mutations affecting inhibition in vitro:** *Reduces sensitivity:* F428A (TM5) |
| C16 ω3 Glycine |  | 810 (460 – 1400) (3) | >10 | 65.3 ± 1.9 | **Mutations affecting inhibition in vitro:** *Reduces sensitivity:* V432A (TM5) |
| C16 ω7 Glycine |  | 1500 (1300 – 1800) (4) | >10 | 92.5 ± 1.6 | **Mutations affecting inhibition in vitro:** *Reduces sensitivity:* F428A (TM5), V432A (TM5) |
| C16 ω11 Glycine |  | 3400 (2500 – 4800) (4) | >10 | 97.9 ± 2.3 | **Mutations affecting inhibition in vitro:** *Unaffected:* F428A (TM5) |
| C14 ω5 Glycine |  | 9200 (7300 – 12000) (3) | >10 | 100 ± 7.2 | **Mutations affecting inhibition in vitro:** *Reduces sensitivity:* F428A (TM5) |

Structures of acyl amino acids and their activity on WT GlyT2 and GlyT1 (previously published)^11,12^ Data presented are mean and 95% confidence intervals or mean ± SEM.

Residues identified by MD was defined by residues that are in contact with the inhibitor after 100 ns of MD simulation, where any heavy atom of the residue was within a distance of <4Å of any atom of the inhibitor.
