# Supplementary material for: Identification of an allosteric binding site on the human glycine transporter, GlyT2, for bioactive lipid analgesics
Source: eLife. 2019 Oct 17;8:e47150. doi: 10.7554/eLife.47150 (PMC6797481; doi:10.7554/eLife.47150)
Supplement: Supplementary file 3. [file elife-47150-supp3.docx]

**Supplementary File 3.** **EC_50_ values for glycine transport of WT and mutant GlyT2 and GlyT1 transporters.**

| **Region** | **Mutation** | **Glycine EC_50_ (µM)** |
| --- | --- | --- |
| **GlyT2** | | |
| **GlyT2 WT** |  | 14.5 ± 2.6 (5) |
| **EL4** | Y550L | 62.2 ± 11.4 (3) (p=0.9718) |
| **TM8** | P561S | 15.5 ± 4.1 (3) (p=0.9999) |
|  | W563L | 4.9 ± 0.8 (3) (p=0.9920) |
|  | F569A/V/L | No transport currents |
|  | L569F | 7.0 ± 0.6 (3) (p=0.9993) |
| **TM5** | R439L | 23.0 ± 4.0 (5) (p=0.9996) |
|  | V432A | 114 ± 21.6 (7) (p=0.9952) |
|  | P429A | No transport currents |
|  | F428A | 6.2 ± 0.4 (3) (p=0.9991) |
| **Vestibule allosteric site** | D469A | 110 ± 12.8 (5) (p=0.9819) |
|  | T472A | No transport currents |
|  | Q473A | 498 ± 146 (5) (p=0.4819) |
|  | T634A | 28.6 ± 3.2 (5) (p=0.9999) |
|  | S696A | 25.6 ± 2.7 (4) (p=0.9999) |
|  | E701A | 16.5 ± 0.87 (5) (p=9997) |
| **GlyT1** | | |
| **GlyT1 WT** |  | 14.4 ± 1.2 (3) |
| **EL4** | I425L | 45.1 ± 9.6 (3) (p=0.5225) |

Glycine concentration response curves were performed on WT and mutant transporters to generate EC_50_ values. Transport currents were measured in the presence of increasing concentrations of glycine (1 – 300 μM). Currents were fit to the modified Michaelis-Menten equation and normalised to the calculated I_max_. A one-way ANOVA determined any difference in mutant GlyT2 affinity compared to WT GlyT2. A two-tailed t test compared mutant (L425I) GlyT1 with WT GlyT1. No EC_50_ values were considered significantly different than WT.
