# Supplementary material for: Identification of an allosteric binding site on the human glycine transporter, GlyT2, for bioactive lipid analgesics
Source: eLife. 2019 Oct 17;8:e47150. doi: 10.7554/eLife.47150 (PMC6797481; doi:10.7554/eLife.47150)
Supplement: Supplementary file 4. [file elife-47150-supp4.docx]

**Supplementary File 4.** Percentage of the total simulation time in which residues are in contact with the lipid inhibitors. Only interactions that occur for >30% of the total simulation time are reported. For OLSer contact residues are only calculated for the simulation in which the lipid inhibitor remains bound in the extracellular allosteric pocket.^a^

|  | OLLeu | OLLys | OLSer | OLTrp |
| --- | --- | --- | --- | --- |
| V214 | 68.99% | 72.85% | 35.01% | 73.25% |
| W215 | 47.04% | - | - | 54.20% |
| P218 | - | 33.71% | - | - |
| P429 | - | 36.58% | - | - |
| L433 | 67.82% | 52.10% | - | - |
| L436 | 89.84% | 65.82% | 94.56% | 84.98% |
| R439 | 99.23% | 75.68% | 61.14% | 99.87% |
| G440 | 63.76% | - | 61.84% | 44.50% |
| L443 | 93.07% | 55.53% | 86.61% | 79.58% |
| P444 | - | - | 53.25% | - |
| T512 | - | 34.34% | - | - |
| A516 | - | 43.67% | - | - |
| F518 | - | - | 36.36% | - |
| V419 | 69.79% | 46.24% | 77.22% | 47.80% |
| I520 | 85.38% | 65.52% | 33.87% | 64.92% |
| S522 | - | - | 64.39% | - |
| V523 | 91.11% | 97.30% | 76.32% | 92.94% |
| F526 | 73.32% | 94.84% | 75.12% | 98.10% |
| M527 | - | 67.39% | - | 77.12% |
| R531 | - | 38.07% | - | 78.71% |
| G542 | 40.54% | - | - | - |
| I545 | 60.93% | 70.42% | - | 65.82% |
| A546 | 55.50% | - | - | - |
| V549 | - | 45.64% | - | 71.52% |
| Y550 | 100.00% | 100.00% | 98.80% | 94.10% |
| A553 | 55.66% | 78.78% | 34.17% | 97.77% |
| L554 | - | 31.35% | 45.55% | 48.87% |
| T555 | - | - | - | 36.38% |
| R556 | - | - | 53.50% | 80.41% |
| L557 | 89.11% | 96.60% | 86.61% | 97.87% |
| P558 | - | - | 56.74% | - |
| W563 | 94.14% | 70.82% | 51.50% | - |
| F567 | 98.87% | 97.34% | 50.85% | 95.47% |
| M570 | 68.32% | 53.23% | - | 37.97% |
| L571 | 46.60% | - | - | 31.15% |
| ^a^A contact is defined as a distance of <4 Å between the residues. | | | | |
